# Supplementary material for: Multiple blood feeding in mosquitoes shortens the Plasmodium falciparum incubation period and increases malaria transmission potential
Source: PLoS Pathog. 2020 Dec 31;16(12):e1009131. doi: 10.1371/journal.ppat.1009131 (PMC7774842; doi:10.1371/journal.ppat.1009131)
Supplement: S1 Text — (PDF) [file ppat.1009131.s001.pdf]

S1 Text

Modeling Details

Our simplified formula for R_0,

$$R_0(T) = \sqrt{f(T)e^{-g(T)}},$$

can be related to the original equation from Mordecai *et al.* [30] reproduced here as,

$$R_0(T) = \sqrt{\frac{a(T)^2 bc(T) e^{-\frac{\mu(T)}{PDR(T)}} EFD(T) p_{EA}(T) MDR(T)}{N r \mu^3(T)}},$$

by

$$f(T) = \frac{a(T)^2 bc(T) EFD(T) p_{EA}(T) MDR(T)}{N r \mu^3(T)}$$

and

$$g(T) = \frac{\mu(T)}{PDR(T)}.$$

The expression is based on temperature-dependent trait data from *Anopheles* species that vary with temperature including biting rate (*a*), vector competence (*bc*), adult mosquito mortality (μ), parasite development rate (*PDR*), egg-to-adult survival probability (p_{EA}), mosquito development rate (*MDR*) and eggs laid per female per day (*EFD*). Note that adult mortality is calculated through daily adult survival (*p*) via $p = e^{-\mu}$; parasite development rate is one over the extrinsic incubation period (*EIP*); and mosquito development rate is one over the larval development time (τ_{EA}). Human related quantities—human density (*N*) and recovery rate (*r*)—are not temperature-dependent. Furthermore, explicit values are not necessary as *N* and *r* cancel out in our final ratios. The temperature-dependent traits of biting rate, parasite development rate and mosquito development time were fit to Brière functions, represented by

$$cT(T - T_0)(T_m - T)^{\frac{1}{2}},$$

where *c*, T_0 and T_m are constants defined in previously published work (**S5 Table**) [30]. The temperature-dependent traits vector competence, daily adult survival, egg-to-adult survival probability and eggs laid per female were fit to quadratic functions, represented by

$$qT^2 + rT + s,$$

where *q*, *r*, and *s* are constants determined in previously published work (**S6 Table**) [30]. The original calculation of this function was done using EFD data from *Aedes albopictus*, as there were no data available describing the relationship between *Anopheles* EFD and temperature. We updated this function using recently published data from Villena *et al*. [61], which we fit to a quadratic function using the nls function in R as in [30] (**S3 Fig**).

Our modified basic reproductive number incorporating a second blood feeding by scaling EIP using the term β, which we refer to as R_0^b given by

$$R_0^b(T) = \sqrt{\frac{a(T)^2 bc(T) e^{-\beta \frac{\mu(T)}{PDR(T)}} EFD(T) p_{EA}(T) MDR(T)}{Nr\mu^3(T)}}.$$

We determine the scaling parameter β as the relative reduction in EIP in the presence of a second blood feed, given by

$$\beta = \frac{2\ blood\ feeds}{1\ blood\ feed} = \frac{8.63}{10.88} = 0.73.$$

The change in R_0 (**S4 Fig**) using a shortened EIP is shown by the ratio of the modified R_0 to the original R_0 as

$$\frac{R_0^b}{R_0}.$$
