# Supplementary figures and images for: Multiple blood feeding in mosquitoes shortens the Plasmodium falciparum incubation period and increases malaria transmission potential
Source: PLoS Pathog. 2020 Dec 31;16(12):e1009131. doi: 10.1371/journal.ppat.1009131 (PMC7774842; doi:10.1371/journal.ppat.1009131)

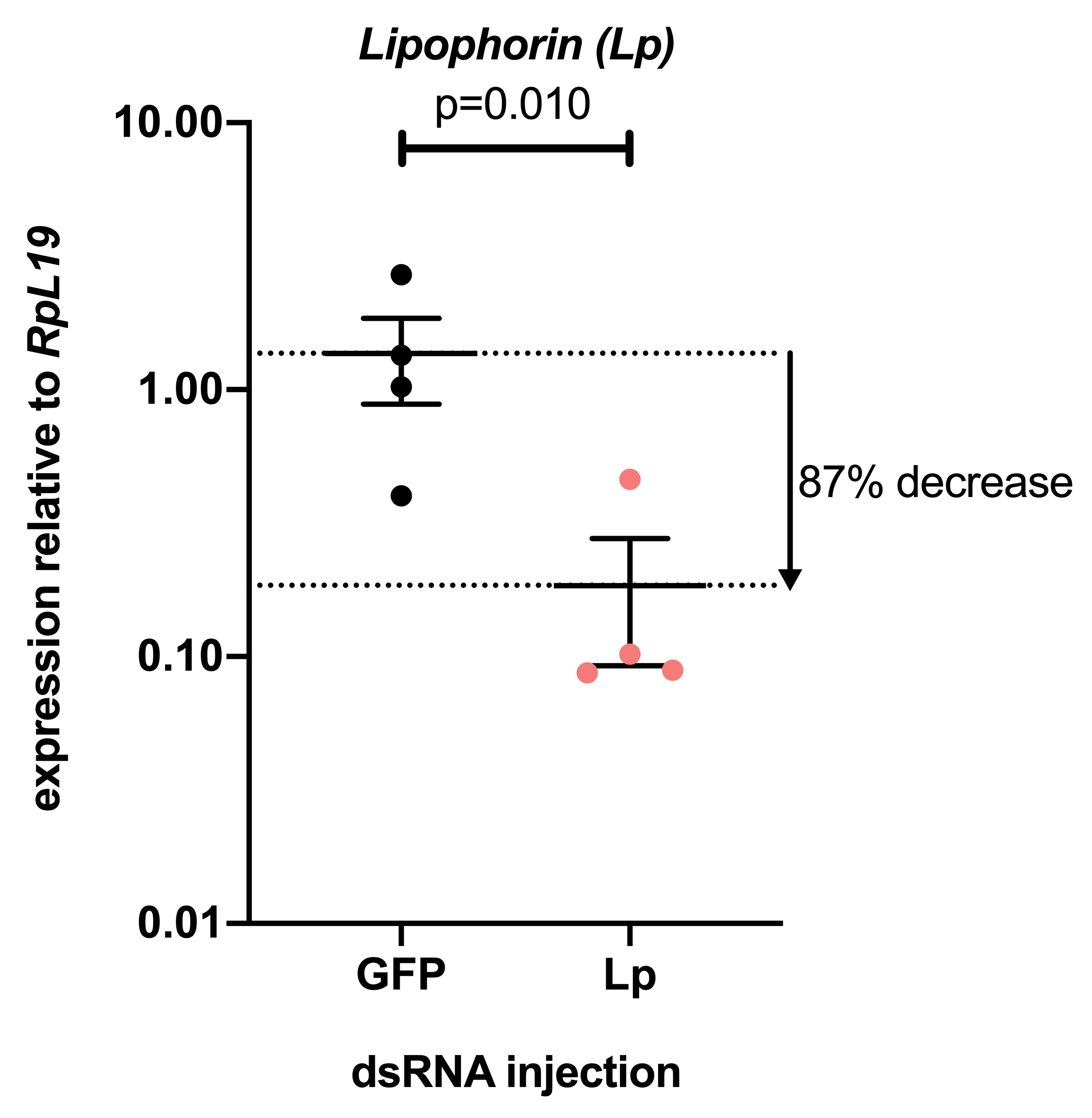

Supplement: S1 Fig — Lp gene expression was determined in pools of 5–10 decapitated females at 6 d post injection (3 d pIBM) at the time of the second blood feed. Lp expression levels were normalized to Rpl19. Four biological replicates were analyzed with means ± standard error shown by horizontal bars. Unpaired t-test. (TIF) [file ppat.1009131.s002.tif]

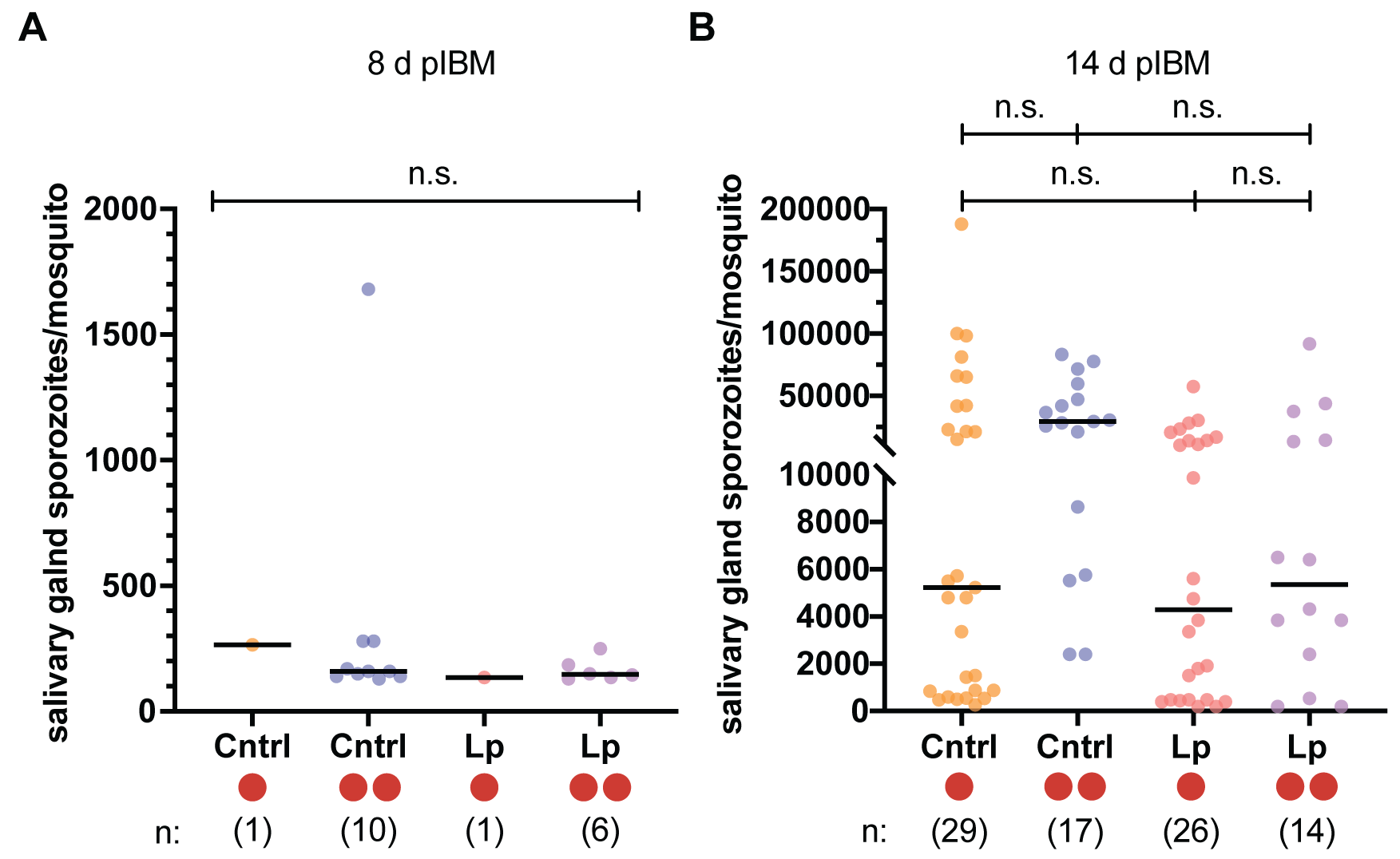

Supplement: S2 Fig — (A) Salivary glands of females fed twice (two red circles) show more sporozoites than females fed once (one red circle) at 8 d but low prevalence in singly-fed groups prevents a determination of statistical significance. (B) Sporozoite levels in salivary glands at 14 d pIBM are comparable between singly and doubly-fed control and Lp-silenced mosquitoes (Linear mixed model; #BF: p = 0.034; dsRNA: p = 0.0053; FDR-corrected post-hoc Student’s t tests shown). Neither the increase in infection intensity across the 2BF groups, nor the decreased sporozoite intensity in Lp-silenced groups persist after post-hoc testing (S1 and S2 Tables). Horizontal bars indicate median values. n = numbers of mosquitoes analyzed from 3 different experiments. n.s. = not statistically significant. (TIF) [file ppat.1009131.s003.tif]

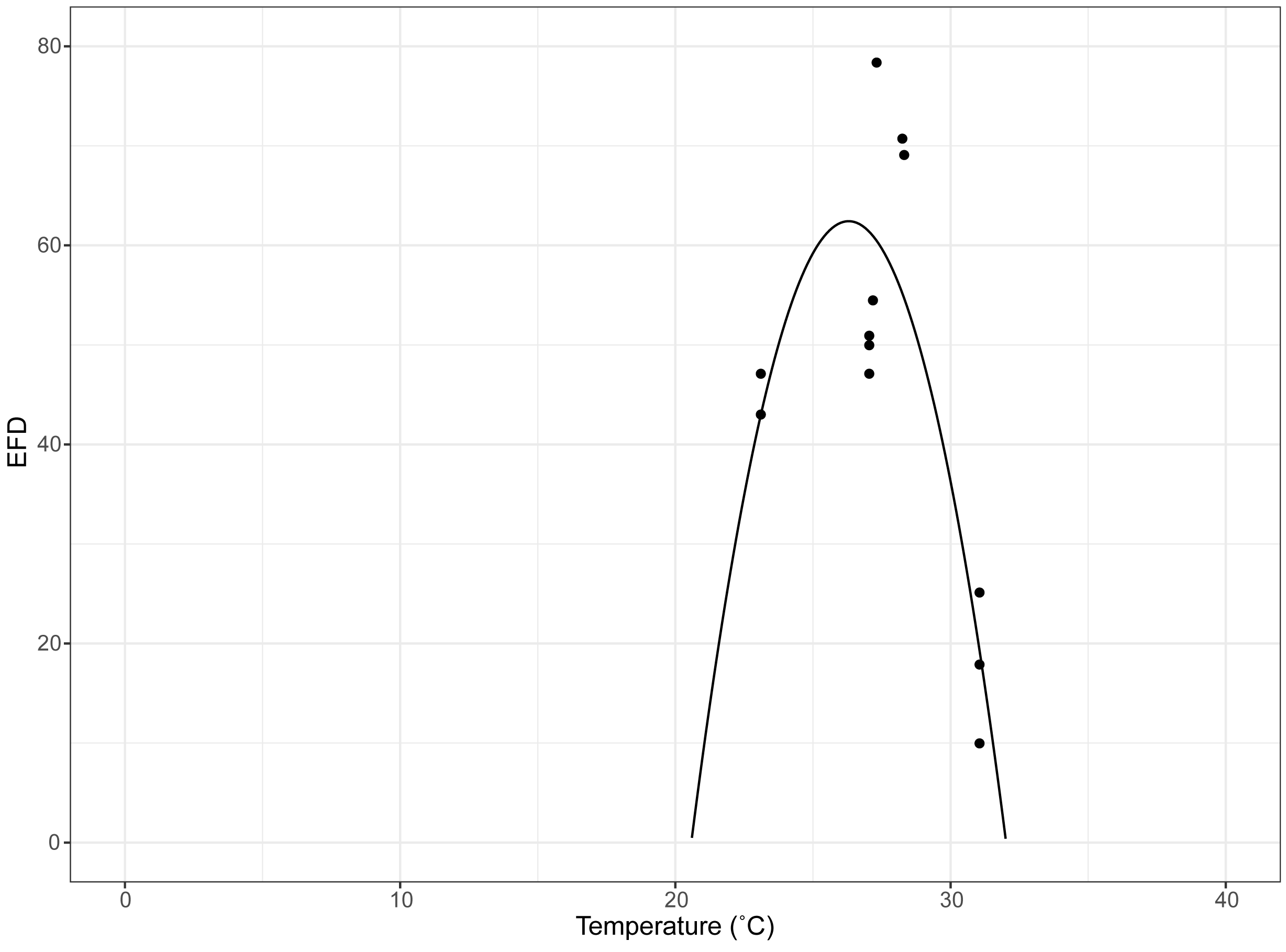

Supplement: S3 Fig — Data points extracted from Villena et al. [61] were fit to a quadratic function using the nls function in R as described in Mordecai et al. [30]. The fitted quadratic function is shown, with parameters listed in S6 Table. (TIF) [file ppat.1009131.s004.tif]

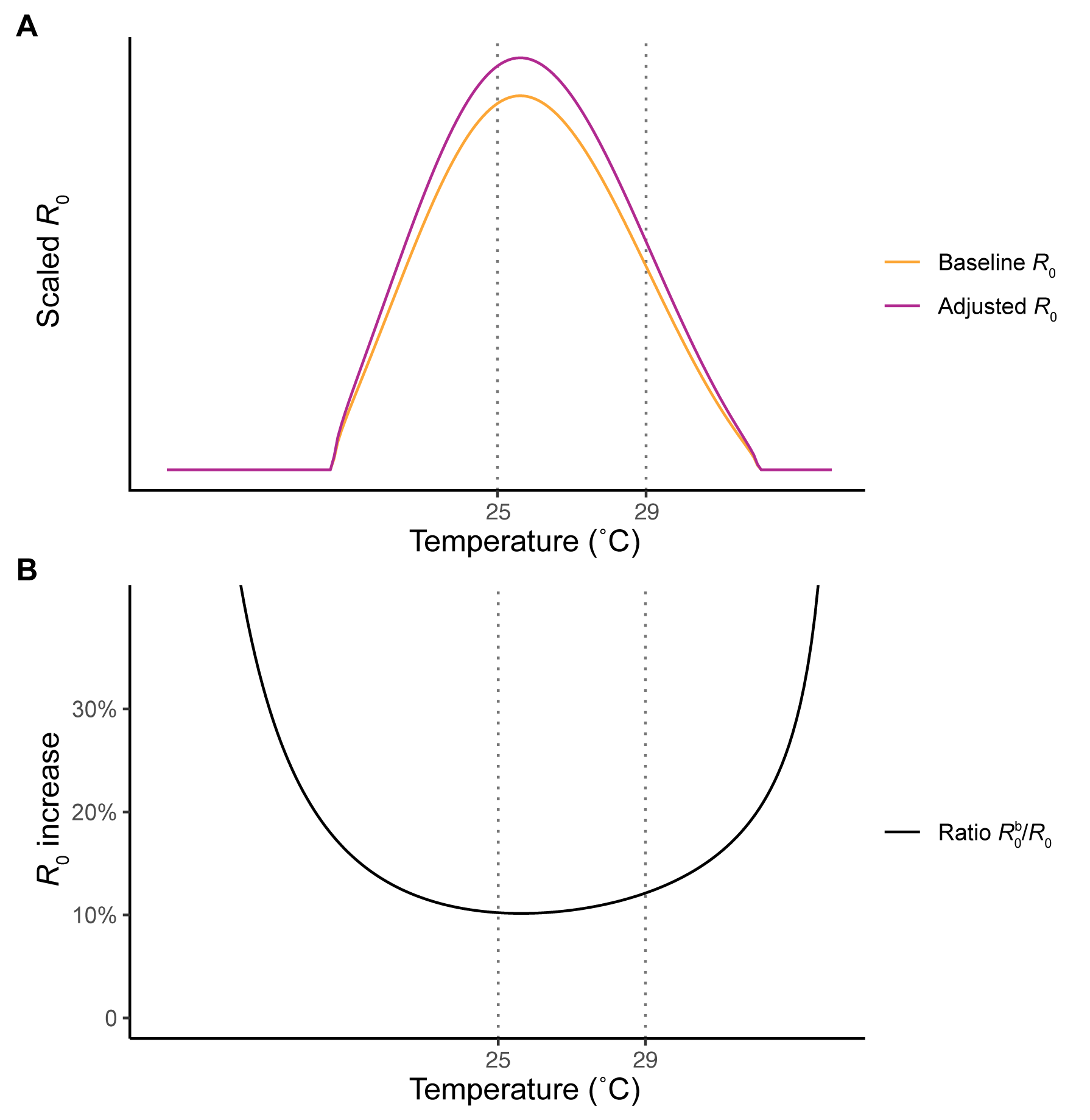

Supplement: S4 Fig — (A) Baseline R0 (orange) and Adjusted R0b (purple) as temperature varies. No numeric scale is given as raw R0 values depend on parameters, such as population size, that are not temperature dependent and cancel out in the R0 ratio. (B) The increase in R0 with shortened EIP as a function of temperature. Within the temperature range 27 ± 2°C, the increase in R0 is between 10.1% and 12.1%. (TIF) [file ppat.1009131.s005.tif]

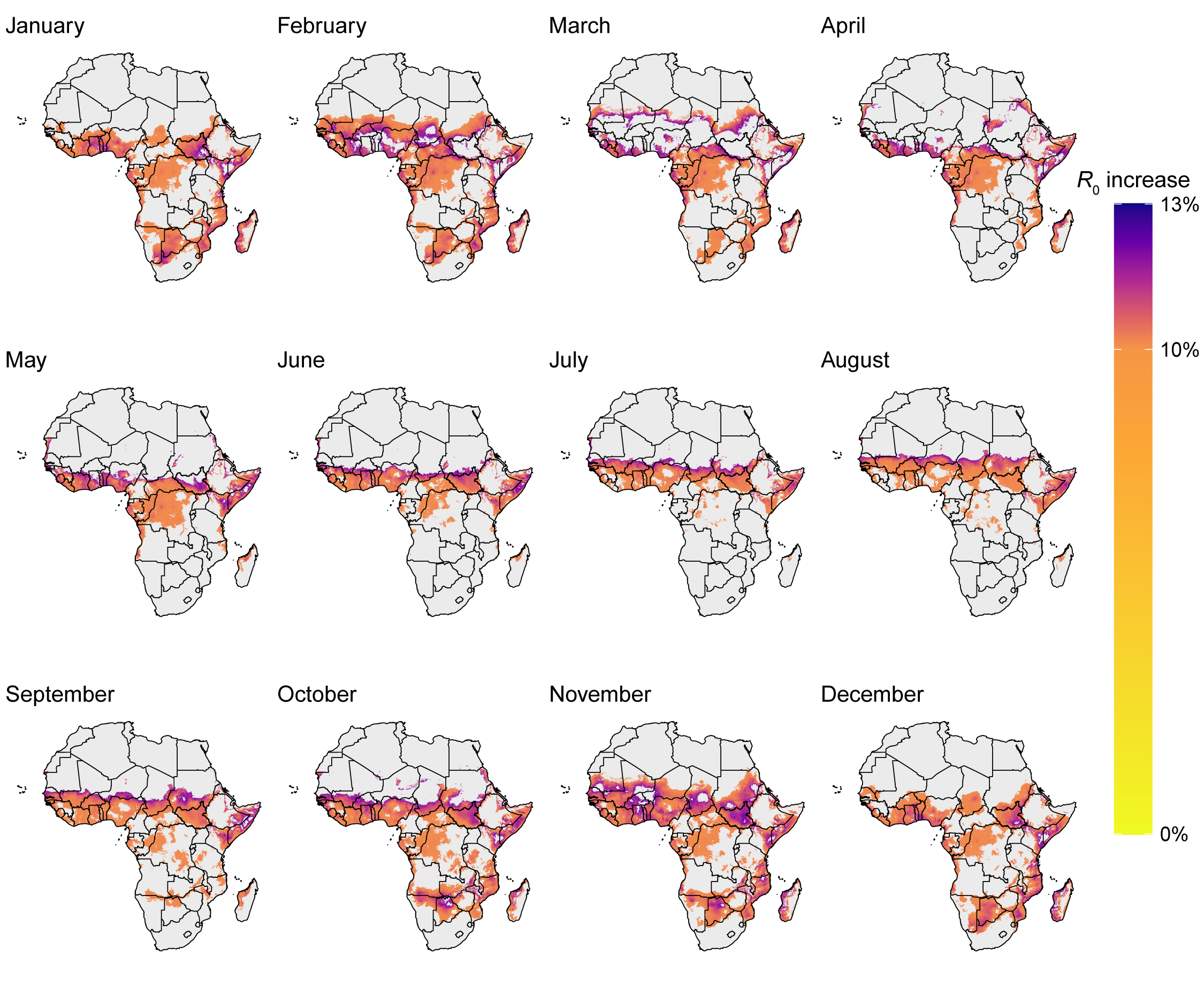

Supplement: S5 Fig — We calculated the monthly changes in R0 for each 5x5 km grid cell by taking the ratio R0b/R0 using the mean temperature of each month that has a mean temperature at 27 ± 2°C. The restricted data points shown here are used to create the summary maps in Fig 4. (TIF) [file ppat.1009131.s006.tif]
