# Supplementary material for: Multiple blood feeding in mosquitoes shortens the Plasmodium falciparum incubation period and increases malaria transmission potential
Source: PLoS Pathog. 2020 Dec 31;16(12):e1009131. doi: 10.1371/journal.ppat.1009131 (PMC7774842; doi:10.1371/journal.ppat.1009131)
Supplement: S1 Table — JMP 14 Pro statistical software was used to construct models for data analysis to account for multiple variables in an experiment. Residual Maximum Likelihood (REML) variance components analysis was used by fitting linear mixed models after cube-root transformation to resemble a normal distribution. The number of blood feeds, dsRNA injection group and their interaction were used as fixed effects and replicate was included as a random effect. Effect test outputs are reported here. Multiple comparisons were calculated using 4 pairwise Student’s t tests followed by FDR correction (see S2 Table). d pIBM = days post infectious blood meal; #BF = number of blood feeds; FDR = false discovery rate. (DOCX) [file ppat.1009131.s007.docx]

**S1 Table**

| **Fig** | **Comparison** | **Test/Model** | **Effect Test Outputs** |
| --- | --- | --- | --- |
| **1B** | 7 d pIBM oocysts, where >0 | Linear Mixed Model  followed by 4 post-hoc t-tests, FDR corrected (**S2 Table**) | #BF p=0·5719  dsRNA p<0.0001  dsRNA x #BF p=0.1733 |
| **1C** | 7 d pIBM  mean oocyst size |  | #BF p<0·0001  dsRNA p=0.1617  dsRNA x #BF p=0.3842 |
| **3B** | 10 d pIBM sporozoites,  where >0 |  | #BF p=0·0001  dsRNA p=0.0822  dsRNA x #BF p=0.9128 |
| **5A** | 7 d pIBM mean oocyst size |  | #BF p<0·0001  genotype p<0.0001  genotype x #BF p=0.0236 |
| **5B** | 7 d pIBM oocysts, where >0 |  | #BF p=0·0825  genotype p<0.0001  genotype x #BF p=0.4823 |
| **5C** | 10 d pIBM sporozoites,  where >0 |  | #BF p<0.0001  genotype p=0.0056  genotype x #BF p=0.2767 |
| **S2A** | 8 d pIBM sporozoites,  where >0 | n/a | Insufficient data to run model |
| **S2B** | 14 d pIBM sporozoites,  where >0 | Linear Mixed Model followed by 4 post-hoc t-tests, FDR corrected (**S2 Table**) | #BF p=0·0336  dsRNA p=0.0053  dsRNA x #BF p=0.4303 |
