# Supplementary material for: Multiple blood feeding in mosquitoes shortens the Plasmodium falciparum incubation period and increases malaria transmission potential
Source: PLoS Pathog. 2020 Dec 31;16(12):e1009131. doi: 10.1371/journal.ppat.1009131 (PMC7774842; doi:10.1371/journal.ppat.1009131)
Supplement: S2 Table — Significant differences in oocyst size and oocyst and sporozoite intensity using an FDR of 0.05. See S1 Table. (DOCX) [file ppat.1009131.s008.docx]

**S2 Table**

| **Fig 1B** | **p-value** | **FDR-adjusted p-value** | **Significant?** |
| --- | --- | --- | --- |
| Cntrl 1BF – Cntrl 2BF | 1.80 x 10^-1^ (0.1799) | 2.40 x 10^-1^ (0.2398) | No |
| Cntrl 1BF – Lp 1BF | 7.72 x 10^-3^ (0.0077) | 1.54 x 10^-2^ (0.0154) | Yes |
| Cntrl 2BF – Lp 2BF | 3.21 x 10^-5^ | 1.28 x 10^-4^ (0.0001) | Yes |
| Lp 1BF – Lp 2BF | 5.67 x 10^-1^ (0.5689) | 5.67 x 10^-1^ (0.5689) | No |
| **Fig 1C** | **p-value** | **FDR-adjusted p-value** | **Significant?** |
| Cntrl 1BF – Cntrl 2BF | 5.45 x 10^-29^ | 1.09 x 10^-28^ | Yes |
| Cntrl 1BF – Lp 1BF | 6.90 x 10^-1^ (0.6896) | 6.90 x 10^-1^ (0.6896) | No |
| Cntrl 2BF – Lp 2BF | 1.28 x 10^-1^ (0.1276) | 1.72 x 10^-1^ (0.1707) | No |
| Lp 1BF – Lp 2BF | 4.06 x 10^-34^ | 1.62 x 10^-33^ | Yes |

| **Fig 3B** | **p-value** | **FDR-adjusted p-value** | **Significant?** |
| --- | --- | --- | --- |
| Cntrl 1BF – Cntrl 2BF | 3.70 x 10^-3^ (0.0037) | 3.04 x 10^-3^ (0.0148) | Yes |
| Cntrl 1BF – Lp 1BF | 2.66 x 10^-1^ (0.2658) | 2.66 x 10^-1^ (0.2658) | No |
| Cntrl 2BF – Lp 2BF | 1.48 x 10^-1^ (0.1478) | 1.97 x 10^-1^ (0.1971) | No |
| Lp 1BF – Lp 2BF | 6.87 x 10^-3^ (0.0069) | 1.37 x 10^-2^ (0.0137) | Yes |

| **Fig 5A** | **p-value** | **FDR-adjusted p-value** | **Significant?** |
| --- | --- | --- | --- |
| Cntrl 1BF – Cntrl 2BF | 9.28 x 10^-17^ | 3.71 x 10^-16^ | Yes |
| Cntrl 1BF – Δ*zpg* 1BF | 1.81 x 10^-9^ | 3.62 x 10^-9^ | Yes |
| Cntrl 2BF – Δ*zpg* 2BF | 2.49 x 10^-3^ (0.0025) | 2.49 x 10^-3^ (0.0025) | Yes |
| Δ*zpg* 1BF – Δ*zpg* 2BF | 2.43 x 10^-8^ | 3.24 x 10^-8^ | Yes |
| **Fig 5B** | **p-value** | **FDR-adjusted p-value** | **Significant?** |
| Cntrl 1BF – Cntrl 2BF | 8.57 x 10^-2^ (0.0857) | 1.14 x 10^-1^ (0.1143) | No |
| Cntrl 1BF – Δ*zpg* 1BF | 1.09 x 10^-7^ | 4.36 x 10^-7^ | Yes |
| Cntrl 2BF – Δ*zpg* 2BF | 8.09 x 10^-6^ | 1.62 x 10^-5^ | Yes |
| Δ*zpg* 1BF – Δ*zpg* 2BF | 4.57 x 10^-1^ (0.4571) | 4.57 x 10^-1^ (0.4571) | No |
| **Fig 5C** | **p-value** | **FDR-adjusted p-value** | **Significant?** |
| Cntrl 1BF – Cntrl 2BF | 2.73 x 10^-5^ | 5.46 x 10^-5^ | Yes |
| Cntrl 1BF – Δ*zpg* 1BF | 2.09 x 10^-2^ (0.0209) | 2.79 x 10^-2^ (0.0279) | Yes |
| Cntrl 2BF – Δ*zpg* 2BF | 1.13 x 10^-1^ (0.1126) | 1.13 x 10^-1^ (0.1126) | No |
| Δ*zpg* 1BF – Δ*zpg* 2BF | 3.14 x 10^-6^ | 1.26 x 10^-5^ | Yes |

| **Fig S2B** | **p-value** | **FDR-adjusted p-value** | **Significant?** |
| --- | --- | --- | --- |
| Cntrl 1BF – Cntrl 2BF | 3.10 x 10^-2^ (0.0310) | 6.20 x 10^-2^ (0.0620) | No |
| Cntrl 1BF – Lp 1BF | 8.74 x 10^-2^ (0.0874) | 1.16 x 10^-1^ (0.1165) | No |
| Cntrl 2BF – Lp 2BF | 2.43 x 10^-2^ (0.0243) | 9.72 x 10^-2^ (0.0972) | No |
| Lp 1BF – Lp 2BF | 3.51 x 10^-1^ (0.3510) | 3.51 x 10^-1^ (0.3510) | No |
