# Supplementary material for: Multiple blood feeding in mosquitoes shortens the Plasmodium falciparum incubation period and increases malaria transmission potential
Source: PLoS Pathog. 2020 Dec 31;16(12):e1009131. doi: 10.1371/journal.ppat.1009131 (PMC7774842; doi:10.1371/journal.ppat.1009131)
Supplement: S3 Table — GraphPad Prism 8 was used for logistic regression, Fisher’s exact and χ2 tests. (DOCX) [file ppat.1009131.s009.docx]

**S3 Table**

| **Fig** | **Comparison** | **Test/Model** | **Test Outputs** |
| --- | --- | --- | --- |
| **1B** | 7 d pIBM oocyst prevalence | χ^2^ test | χ^2^=4.263  d.f.=3  p>0·05 |
| **3A** | 7 d pIBM sporozoite prevalence |  | χ^2^=5.697  d.f.=3  p>0·05 |
|  | 8 d pIBM sporozoite prevalence | χ^2^ test  followed by 4 post‑hoc χ^2^ tests, FDR corrected  (**S4 Table**) | χ^2^=15.01  d.f.=3  p=0·0018 |
|  | 10 d pIBM sporozoite prevalence |  | χ^2^=43.33  d.f.=3  p<0·0001 |
|  | 14 d pIBM sporozoite prevalence | χ^2^ test | χ^2^=2.445  d.f.=3  p>0·05 |
| **3C** | 7–14d pIBM sporozoite prevalence | Logistic regression  Lines of best fit: | Cntrl 1BF: log odds =  -8·858+0·8142*d  Cntrl 2BF: log odds =  -8·646+1·002*d  Lp 1BF: log odds =  -8.228+0.7125*d  Lp 2BF: log odds =  -8.762+0.9520*d |
|  |  | EIP_50_ ± s.e.  Z test | Cntrl 1BF: 10.88 ± 0.32 d  Cntrl 2BF: 8.63 ± 0.23 d  Lp 1BF: 11.55 ± 0.37 d  Lp 2BF: 9.20 ± 0.32 d  Cntrl 1BF–Cntrl 2BF: z=5.74  Cntrl 1BF–Lp 1BF: z=-1.36  Cntrl 2BF–Lp 2BF: z=-1.74  Lp 1BF–Lp 2BF: z=5.28 |
| **5B** | 7 d pIBM oocyst prevalence | χ^2^ test | χ^2^=6.488  d.f.=3  p>0·05 |
| **5C** | 10 d pIBM sporozoite prevalence | χ^2^ test,  followed by 4 post‑hoc χ^2^ tests, FDR corrected  (**S4 Table**) | χ^2^=83·41  d.f.=3  p<0·0001 |
|  | 10 d pIBM sporozoite prevalence  (pooled by genotype) | Fisher’s exact test | p<0·0001 |
