# Supplementary material for: Multiple blood feeding in mosquitoes shortens the Plasmodium falciparum incubation period and increases malaria transmission potential
Source: PLoS Pathog. 2020 Dec 31;16(12):e1009131. doi: 10.1371/journal.ppat.1009131 (PMC7774842; doi:10.1371/journal.ppat.1009131)
Supplement: S4 Table — Significant differences in infection prevalence using an FDR of 0.05. See S3 Table. (DOCX) [file ppat.1009131.s010.docx]

**S4 Table**

| **Fig 3A – 8 d pIBM** | **p-value** | **FDR-adjusted p-value** | **Significant?** |
| --- | --- | --- | --- |
| Cntrl 1BF – Cntrl 2BF | 2.56 x 10^-3^ (0.0026) | 1.04 x 10^-2^ (0.0104) | Yes |
| Cntrl 1BF – Lp 1BF | 1.00 | 1.00 | No |
| Cntrl 2BF – Lp 2BF | 3.85 x 10^-1^ (0.3848) | 5.13 x 10^-1^ (0.5131) | No |
| Lp 1BF – Lp 2BF | 5.17 x 10^-3^ (0.0517) | 1.03 x 10^-1^ (0.1034) | No |
| **Fig 3A – 10 d pIBM** | **p-value** | **FDR-adjusted p-value** | **Significant?** |
| Cntrl 1BF – Cntrl 2BF | 2.19 x 10^-6^ | 8.76 x 10^-6^ | Yes |
| Cntrl 1BF – Lp 1BF | 3.29 x 10^-1^ (0.3289) | 3.29 x 10^-1^ (0.3289) | No |
| Cntrl 2BF – Lp 2BF | 1.69 x 10^-1^ (0.1687) | 2.23 x 10^-1^ (0.2253) | No |
| Lp 1BF – Lp 2BF | 2.10 x 10^-5^ | 4.20 x 10^-5^ | Yes |
| **Fig 3C – ΔEIP_50_** | **p-value** | **FDR-adjusted p-value** | **Significant?** |
| Cntrl 1BF – Cntrl 2BF | 9.47 x 10^-9^ | 3.79 x 10^-8^ | Yes |
| Cntrl 1BF – Lp 1BF | 1.74 x 10^-1^ (0.1740) | 1.74 x 10^-1^ (0.1740) | No |
| Cntrl 2BF – Lp 2BF | 8.19 x 10^-2^ (0.0819) | 1.09 x 10^-1^ (0.1092) | No |
| Lp 1BF – Lp 2BF | 1.29 x 10^-7^ | 2.58 x 10^-7^ | Yes |

| **Fig 5C** | **p-value** | **FDR-adjusted p-value** | **Significant?** |
| --- | --- | --- | --- |
| Cntrl 1BF – Cntrl 2BF | 1.42 x 10^-11^ | 5.68 x 10^-11^ | Yes |
| Cntrl 1BF – Δ*zpg* 1BF | 5.74 x 10^-9^ | 1.15 x 10^-8^ | Yes |
| Cntrl 2BF – Δ*zpg* 2BF | 1.52 x 10^-1^ (0.1517) | 1.52 x 10^-1^ (0.1517) | No |
| Δ*zpg* 1BF – Δ*zpg* 2BF | 2.76 x 10^-3^ (0.0028) | 3.68 x 10^-3^ (0.0037) | Yes |
