# Supplementary material for: Multiple blood feeding in mosquitoes shortens the Plasmodium falciparum incubation period and increases malaria transmission potential
Source: PLoS Pathog. 2020 Dec 31;16(12):e1009131. doi: 10.1371/journal.ppat.1009131 (PMC7774842; doi:10.1371/journal.ppat.1009131)
Supplement: S5 Table — All parameters from Mordecai et al. [30]. See references within. (DOCX) [file ppat.1009131.s011.docx]

**S5 Table**

| **Trait** | ***c*** | ***T_0_* (°C)** | ***T_m_* (°C)** |
| --- | --- | --- | --- |
| *a* | 0.000203 | 11.7 | 42.3 |
| *PDR* | 0.000111 | 14.7 | 34.4 |
| *MDR* | 0.000111 | 14.7 | 34.0 |
