# Supplementary material for: Multiple blood feeding in mosquitoes shortens the Plasmodium falciparum incubation period and increases malaria transmission potential
Source: PLoS Pathog. 2020 Dec 31;16(12):e1009131. doi: 10.1371/journal.ppat.1009131 (PMC7774842; doi:10.1371/journal.ppat.1009131)
Supplement: S6 Table — Parameters for bc, p, and pEA from Mordecai et al. [30]. See references within. Parameters for EFD were fit to data published in Villena et al. [61]. (DOCX) [file ppat.1009131.s012.docx]

**S6 Table**

| **Trait** | ***q*** | ***r*** | ***s*** |
| --- | --- | --- | --- |
| *bc* | -0.54 | 25.2 | -206.0 |
| *p* | -0.000828 | 0.0367 | 0.522 |
| *p_EA_* | -0.00924 | 0.453 | -4.77 |
| *EFD* | -1.908 | 100.353 | -1257.111 |
