# Supplementary material for: Multiple blood feeding in mosquitoes shortens the Plasmodium falciparum incubation period and increases malaria transmission potential
Source: PLoS Pathog. 2020 Dec 31;16(12):e1009131. doi: 10.1371/journal.ppat.1009131 (PMC7774842; doi:10.1371/journal.ppat.1009131)
Supplement: Attachment — Submitted filename: Letter to Reviewers.docx [file ppat.1009131.s014.docx]

Boston, October 27, 2020

Dear Editor,

We are pleased to submit our revised manuscript entitled “**Multiple blood feeding in mosquitoes shortens the *Plasmodium falciparum* incubation period and increases malaria transmission potential**” on behalf of our coauthors for consideration for publication in *PLoS Pathogens*. We are grateful for the careful review that our original manuscript received. We have addressed all reviewers’ comments and hope that with these revisions our work will be now acceptable for publication in *PLoS Pathogens*.

Please find below our detailed response to reviewers’ comments:

**Part I - Summary**

Reviewer #1: This is an interesting paper describing how two blood feeds of Anopheles gambiae mosquitoes fastens the maturation of Plasmodium falciparum sporozoites and hence could lead to more rapid transmission of malaria. They provide a model that goes in this direction and also discuss how current efforts in generating transgenic mosquitoes aimed at mosquito control could be affected by the authors finding. The experiments are well executed and described and I have only comments on the writing / data presentation.

Reviewer #2: This is an excellent and potentially very influential piece by the groups of Childs and Catteruccia. As they have done before, they build upon anecdotal knowledge (here: that a 2nd blood meal accelerates or synchronizes sporozoite development) and provide an evidence base and sketch the potential implications for our understanding of transmission. I have mostly minor comments and requests for clarification.

Reviewer #3: This is an important and interesting study with appealing results. The authors have encapsulated well the research question they raised in this study. The experiment, methods and modelling have been given with clarity, and relevant important assumptions are also clearly explained in detail. The results of simple yet powerful epidemiological model of malaria transmission potential in context of R0 have been supported well with experimental evidences and borrow strength from previous studies. The paper highlights important issues related to the current understanding of malaria transmission potential under simplified assumptions that could lead to an underestimation of R0. The findings of the study are important for current and future modelling of malaria dynamics and for control interventions.

The results of the paper are novel regarding the deepened understanding of how blood feeding behaviour and rate influence the vectorial capacity and likewise – R0 – of malaria. The message it disseminates clear and valuable. That is, the parasite EIP is an important parameter in mosquito models and previous studies have overlooked and do not account for multiple blood feeding scenarios to estimate parasite EIP. The study could set a base for future studies about the similar parasites of different mosquitoes and their variants, and the study will likely motivate many studies onwards to incorporate blood feeding rate in vectorial capacity.

The authors are aware of study limitations and have nicely described by proposing further exploration for future work to other Anopheles-Plasmodium combinations. Clearly the model and results of R0 are sensitivity to the parameters and may change with a set of new parameterization. The ones used are good picks but of course subject to uncertainty over time and space. On the other hand, relating directly to the published study by Mordecai and is of course of value, and makes it easier to the added value of this study.

Overall, I find this is an interesting and important scientific contribution which will have scientific and policy impact.

**Part II – Major Issues: Key Experiments Required for Acceptance**

Reviewer #1: No experiments but I think the following two suggestions will help the authors improve their manuscript:

the authors often report the mean in figures, but the average is not informative in non-normally distributed data, please replace with median in figures and text and state in figure legends.

We agree that the median is more appropriate to describe non-normally distributed data. We have changed Fig 1C and Fig 5A to show the median values and described the horizontal bars as medians in the figure legends.

One major difference in transmission the authors data suggests but the authors do not comment about is that there might be ab increased number of mosquitoes harbouring very high numbers of sporozoites in the two blood meal groups, e.g. see Figure 3B, where there seems to be at least a 5 fold increase in the numbers of highly infected mosquitoes. The authors should indicate this in the results section and discuss it in light of recent laboratory single bite transmission experiments by the Sinnis lab which suggested that there might be.a threshold for efficient transmission. The effects seen with increased number of sporozoites early in salivary glands might indeed be much larger if those few high infecting mosquitoes are indeed the primary driver of transmission, as so often also seen in other infectious diseases.

We thank the reviewer for pointing this out. We have now have emphasized in the results that greater numbers of highly infected mosquitoes are present at 10d pIBM in the two blood meal group, and have added a sentence to the discussion (and relative reference from Aleshnick et al., PLoS Pathogens 2020) to highlight this point.

Reviewer #2: The only element of the work I feel uncomfortable with is the extrapolation of findings to EIR estimates. The most widely used maps for estimating relative transmission intensity are based on parasite prevalence estimates in children (e.g. the maps generated by MAP). Clearly those maps are not going to change based on the current findings and if there are reasons to suspect sporogonic development has not been adequately captured in leading transmission models, clearly some other factors should also be wrong since these models (e.g. the Imperial Model and IDM model) do capture transmission with reasonable precision. I wonder whether this part should be in this manuscript. It underlines the importance of the findings but at the same time leaves me with the impression that the authors to some extent oversell their (definitely relevant) finding. Figure 3C is very strong and convincing and already gives a sense of urgency to the findings, figure 4 is less convincing.

Thank you for your comment. We did not wish to oversell our data, or to imply that maps generated by MAP are wrong. Merely, we want to stress the potential significance of mosquito multiple feeding behavior to future modelling studies. In particular, models that *directly incorporate* currently accepted EIP estimates (traditionally taken from a single blood feed) likely systematically misestimate transmission. We have now made this concept clearer in the discussion and have toned down the language to describe the implications of our findings (e.g. removed ‘significantly’ from line 249, and elsewhere).

We agree that a reassessment of other parameters is warranted in light of these findings, particularly mosquito survival, which is intimately connected to EIP.

Reviewer #3: None

**Part III – Minor Issues: Editorial and Data Presentation Modifications**

Reviewer #1: Please use sensible digits when reporting numbers, e.g. 2.25 +/- 0.39 should be reported as 2.3 +/- 0.4, 20.7% should read 21% etc

We have adjusted our reported digits to 2 significant figures, with the exception of the model, where it is appropriate to detail the mean change and range to 3 significant figures (10.5 [10.1 to 12.1]) to avoid implying a symmetrical distribution (11 [10 to 12]).

Please indicate in figure legends how many times an infection was performed and that the n corresponds to mosquitoes analysed, e.g. lines 471, 492 etc: replace ‘sample size’ with ‘numbers of mosquitoes analysed from X different experiments’. This info is better placed in the figure legend then in the materials where the authors state that at least 3 infections were performed.

We have added this information to the figure legends.

Line 134: does size specify volume or area? Please specify

We have specified oocyst area.

Figure 3B: maybe indicate on y-axis that these are salivary gland derived or infectious sporozoites?

We have indicated these sporozoites are present in the salivary glands on the y-axis. We have also added this label to Fig S2 for consistency.

Figure 3C: maybe indicate on y-axis that these are infectious mosquitoes

We have indicated the proportion of mosquitoes that are infectious on the y-axis.

Reviewer #2: It might be worth mentioning that, based on the findings by Ponnudurai, also groups that perform experimental infections typically provide a 2nd blood meal to mosquitoes to ensure adequate and timely sporozoite development.

Thank you for alerting us to this omission. We have mentioned this usage and added references in the introduction.

The statement on the average life-span of mosquitoes is crucial for understanding the importance of the findings but may actually be an over-estimate of average mosquito life spans and is based on two older studies. Another old reference (M. W. Service and H. Towson, “The Anopheles vector,” in Essential Malariology, D. A. Warrell and H. M. Gilles, Eds., pp. 59–84, Arnold, London, UK, 2002) actually indicates 10-14 days as average.

We have added this reference and refined our lifespan estimate to 10–20 days, which further supports our case for the importance of our work, as multiply-feeding mosquitoes are more likely to be capable of parasite transmission within a shorter lifespan.

The statement in lines 185-186 that oocyst size is a good proxy EIP seems too strong. There is considerable variation in oocyst size and the authors do not present evidence that this variation also contributions to variation in EIP. Is there a minimum oocyst size that, with high 'positive predictive value' predicts completion of EIP within a certain period?

We concede that we have not shown in this manuscript that variation in oocyst size contributes to variation in EIP, and have therefore measured our statement to state that larger oocyst sizes are associated with shorter EIP estimates. In our previous work observing oocyst size over time (Fig. 5, Werling, Shaw, Itoe et al., Cell 2019), we observe a threshold for oocyst bursting at approximately 1800µm^2^, and earlier reaching of this threshold was associated with a shorter EIP.

The implications of the findings for mosquitoes with impaired egg development is strong and important.

We agree and thank the reviewer for supporting the concerning implications of these results to proposed vector control strategies.

In the discussion section, I do not fully grasp what the authors intend to say with line 284 'However, the overall impact of additional... feeding frequency'. It seems rather obvious that the prevalence of malaria in a population is important for malaria.

We agree with the reviewer that the sentence was confusing and have now modified it to make our meaning clearer.

The capelin out of heterogeneities (lines 305-310) needs further clarification.

We have rewritten this part to explain more clearly that any variations that may impact *R*_0_ are expected to impact the *R*_0_ estimate under a single or multiple blood feeds equally in a given location, and will thus cancel out when we make the ratio calculation.

Figures are clear and relevant.

Reviewer #3: • Since the paper is a mix of experimental and theoretical approaches and will most likely attract readers of both backgrounds, it would be better if the full expression for ‘ds RNA’ (line 120), ‘dsGFP’ (line 121) is also written as it has been consistently done for other terminologies in the paper.

We have expanded these expressions in their first instance.

• At line 121, (Fig S1) should explicitly refer to supplementary information

We have added this reference to the supplementary information.

• In modelling section, line 413: ‘on’ should be removed from “g(T) depends on linearly on EIP”.

We have made this correction.

• Line 429; the accuracy of the temperature considered in model for sub-Saharan Africa could be elaborated. Temperature data of recent most decades could have been considered too. Is there any patent reason to choose the mean temperature from 1970-2000 (line 429)? It needs some explanation as the relevance of results is tallied with current population (2020) of that region and since temperature is changing rapidly.

We have updated our data source, and are now using the mean temperature for each month over the period 2010-2018, which is the latest year available at the correct resolution from TerraClimate, a publicly available data set that has modeled temperature data separately for each month and year at the correct resolution. As we only consider a range of temperatures around the experimental conditions (27 ± 2 ˚C), the new model results (also including the changes to EFD, see next bullet) are very similar to the previous results, with identical mean 10.5 and range [10.1–12.1].

• In supplementary material, Table S6, model parameter EFD, for instance represents the eggs laid per adult female ‘aedes albopictus’ per day. If this differs from EFD of malaria mosquitoes it can be replaced.

There was not previously sufficient data available describing this parameter’s relationship to temperature in *Anopheles.* However, we identified EFD data from *Anopheles* from a recent preprint (Villena *et al.*, bioRxiv 2020. p. 2020.07.08.194472) and have fit that and updated the function we used in our model.

The new results, after adjustments to the function using updated EFD data and adjustments to the climate data input, are very similar to the previous results, with mean 10.5 and range [10.1–12.1] unchanged. Across the mapped region, the estimated *R*_0_ ratio changes no more than 2% (0.02) in either direction, as shown in the map below.

• I found the change in R0 in relation to the number of months per year of suitable conditions of interest. It appears there is perhaps a relationship there that could be explored further.

We agree that there may be additional relationships to explore. However, we do not wish to overinterpret our data and simplified model at this time, and consider that future work to capture variation in EIP across of range of different temperatures is necessary to gain a better picture of the impact of multiple blood feeding across sub-Saharan Africa.

We thank you for considering our manuscript for publication in *PLOS Pathogens*.

Dr. Flaminia Catteruccia

Professor of Immunology and Infectious Disease

Harvard T. H. Chan School of Public Health

fcatter@hsph.harvard.edu

tel: +1 617 432 1773

Dr. Lauren M. Childs

Assistant Professor of Mathematics

Virginia Tech

lchilds@vt.edu

tel: +1 540 231 8265
